# Influence of Analytic Processing on Divergent and Convergent Thinking Tasks: The Role of Rational and Experiential Thinking Styles

**DOI:** 10.3390/jintelligence11020023

**Published:** 2023-01-21

**Authors:** Jianati Hongdizi, Yu-Xin Cui, Xiang Zhou, Hong-Kun Zhai

**Affiliations:** 1 College Student Mental Health Counseling Center, Xinjiang Medical University, Urumqi 830011, China; 2 Zhou Enlai School of Government, Nankai University, Tianjin 300350, China

**Keywords:** divergent thinking, convergent thinking, analytic processing, thinking styles

## Abstract

Scientific interest in the relationship between analytic processing and creativity has increased in recent years. However, there is conflicting evidence on whether analytic processing reduces or enhances creativity. We hypothesize that differences in creativity measurement paradigms (divergent or convergent thinking tasks) and the research orientation of analytic processing (dispositional or situational) may explain the conflicting findings. The present study aims to investigate how priming analytic processing affects individuals’ performance on divergent and convergent thinking tasks and the moderating role of thinking styles. In Study 1 (*N* = 155), participants were assigned to either an analytic processing group or a control group and performed convergent thinking (Remote Associates Task) and divergent thinking (Alternative Uses Test) tasks after priming. In Study 2 (*N* = 119), we conducted a priming paradigm of analytic processing that differed from Study 1, and a personal experiential-rational thinking style was introduced as a moderator. Results showed that priming analytic processing promoted convergent thinking performance but decreased fluency and flexibility scores on the divergent thinking task (Study 1). Notably, the effect of priming analytic processing on divergent thinking performance was significant only for participants with higher levels of rational thinking style (Study 2). These results suggest that thinking styles and dimensions of creativity should be considered in the relationship between analytic processing and creativity.

## 1. Introduction

Creativity, as an advanced expression of human intelligence, is often defined as the ability to develop novel and practical ideas or products (Sternberg and Lubart 1996). It is an important contributor to political, economic, and cultural development (Sternberg and Kaufman 2010). The study of factors that influence, cultivate, and stimulate creativity has become an enduring research focus in many disciplines, including psychology, education, management, and physiology. The role of cognitive factors has received much attention, and extensive research has been conducted on the predictive role of intelligence, executive functions, and creative self-efficacy on individual creativity (Frith et al. 2021; Batey et al. 2010; Wang et al. 2014).

Analytic processing is a crucial cognitive factor and refers to a form of information processing that is based on consciousness and rules, is driven by reason, consumes more cognitive resources, and is slower (Stanovich and West 2000). It is closely related to individual beliefs, cognition, and behavior (Epley and Gilovich 2006; Wen et al. 2020; Gervais and Norenzayan 2012). A growing number of researchers have studied the relationship between analytic processing and creativity, but their findings vary widely (Pennycook et al. 2015). For example, some studies have shown a significant negative correlation between analytic processing and creativity (Deshayes et al. 2021), suggesting that analytic processing may be detrimental to a person’s ability to generate original ideas (Nijstad et al. 2010). However, other studies have shown that rational thinking can significantly and positively predict individual performance on verbal and figural creativity tasks (Palmiero et al. 2020).

Considering that creativity involves both divergent thinking (generating multiple ideas or solutions) and convergent thinking (searching for the best single solution) (Leung et al. 2012), the conflicting results of previous studies may reflect the differential effects of analytic processing on the two components of creativity. Moreover, there are two important research directions in information processing: dispositional and situational. The former focuses on the temporary activation of analytic or intuitive processing through experimental manipulations, whereas the latter focuses on people’s thinking styles, i.e., individual differences in the extent to which people rely on each type of processing. Previous research on the relationship between creativity and analytic processing has typically focused on only one of these orientations, without considering how thinking styles (information processing tendencies) and situational analytic processing priming jointly influence creative task performance. Therefore, based on dual process theory, the present study sought to examine the effects of analytic processing on two types of creativity task performance (divergent/convergent) through experimental priming and the moderating role of individual’s inherent thinking styles to suggest a possible explanation for previous inconsistent findings and to provide theoretical support for the role of analytic processing in the cultivation of creativity.

## 2. Literature Review

### 2.1. Dual Process Theory

Dual process theory states that two independent information-processing systems are involved in human thinking and reasoning. System 1 (also known as intuitive processing or experiential system) is characterized by rapid processing speed, automatic responses, and parallel processing that requires few psychological resources. System 2 (also known as analytic processing or rational system) is characterized by slow, serial, and controlled processing that follows the rules of reasoning and requires more psychological resources (Stanovich and West 2000; Evans and Stanovich 2013; Norris and Epstein 2011). When System 2 (analytic processing) is activated and cognitive resources are available, System 1 (intuitive processing) input is often overridden (Evans and Stanovich 2013; Kahneman and Frederick 2002). Previous studies have shown that analytic processing can be temporarily boosted by experimental manipulations, and common priming paradigms include visual priming (Gervais and Norenzayan 2012), verbal fluency tasks with scrambled sentences (Gervais and Norenzayan 2012; Swami et al. 2014), writing exercise, etc. (Shenhav et al. 2012).

However, some researchers have also focused on individuals’ relatively stable preferences for these two types of processing (Phillips et al. 2016). Researchers such as Epstein extended the dual process theory to the domain of personality and cognitive style, and suggested that in the problem-solving and decision-making process, there are relatively stable individual differences in people’s reliance on each type of information processing, and people’s general preferences in information processing can be reflected by the concept of thinking style (Epstein et al. 1996). The experiential thinking style reflects dependence on intuitive processing (assigned to System 1 processing), while the rational thinking style reflects dependence on analytic processing (mapping onto System 2 processing) (Epstein et al. 1996; Sowden et al. 2015; Leikas et al. 2007). The point of view of Epstein et al. has been confirmed in many areas of research (Pacini and Epstein 1999; Ares et al. 2014). The present study intends to comprehensively examine the effects of analytic processing priming and individual thinking styles on creativity.

### 2.2. Divergent and Convergent Thinking

Creativity manifests itself in scientific invention, artistic creation, entrepreneurial innovation, and many other areas, and its construct is thought to include two measurable cognitive components: divergent and convergent thinking (Guilford 1967; W. Zhang et al. 2020; Runco and Jaeger 2012). Divergent thinking is an open-ended psychological process that emphasizes unconventionality, searching for variation, and exploring answers from different aspects, whereas convergent thinking emphasizes finding a single solution through analysis and deductive reasoning (Zmigrod et al. 2015). Generally, creativity can be achieved based on a combination of divergent and convergent thinking (Childs et al. 2022). Two prominent measures used in creativity research are the Alternative Uses Task (AUT, Guilford 1967) and the Remote Associates Task (RAT, Mednick 1962), which are most commonly used to assess divergent and convergent thinking, respectively (Cortes et al. 2019). In the AUT, participants are asked to find as many uses as possible for some common objects (e.g., brick, newspaper) so that they can consider and solve problems from different perspectives, and is thus diagnostic of divergent thinking. AUT performance was also found to be a significant predictor of creativity in the product design industry (Kwon et al. 2017). In contrast, RAT asks participants to find a word that can be combined with or is related to each of three seemingly unrelated words. In this task, there is only one correct answer for each item, and it requires fairly tight top-down constraints, making it diagnostic of convergent thinking (Chermahini and Hommel 2010). In addition, Lee et al. (2014) found that the RAT score correlated significantly and positively with scores on several tests of convergent thinking (e.g., fluid intelligence, working memory, academic achievement), indicating favorable external validity and that it correlated only weakly with tests of divergent thinking.

Although both divergent and convergent thinking are important components of creativity, some studies suggest that they are based on different cognitive processes (Hommel et al. 2011). Convergent thinking may rely on strong top-down cognitive control to focus attention on a problem and exclude extraneous information. Divergent thinking, on the other hand, can benefit from a low intensity of control from above to flexibly and quickly shift from one point of view to another (Colzato et al. 2012). Studies have found that several variables, such as meditation (Colzato et al. 2012), personality (Chamorro-Premuzic and Reichenbacher 2008), and risk-taking (Shen et al. 2018) have differential effects on performance in divergent and convergent thinking. 

### 2.3. Analytic Processing, Thinking Styles, and Creativity 

There are differing views in the scientific community about the relationship between analytic processing and creativity. Some researchers believe that analytic processing is not conducive to creativity and that a more intuitive, less analytical approach tends to enhance individual creativity performance (Wiley and Jarosz 2012; Aiello et al. 2012) because intuitive processing can generate a large number of new ideas by association, whereas analytic processing based on linear processing cannot (Sowden et al. 2015). Garfield et al. (2001) used groupware-based techniques to manipulate the cognitive process of creativity development and found that, compared to individuals who used analytic techniques, those who used intuitive problem-solving techniques presented more novel and paradigm-breaking ideas. In contrast, some researchers have argued that analytic processing plays a critical role in the creative process (Kaufmann and Vosburg 1997), and high-level creative problem-solving requires a rational and “rigorous information-processing model” (Rothenberg 1990). The Creativity Diamond Framework (Childs et al. 2022) considers analytic thinking as an important tool for fostering creativity. Barr et al. (2015) also confirmed a positive relationship between analytic processing and RAT creative task performance. 

Due to the insufficient and contradictory results, it is necessary to further investigate the relationship between creativity and analytic processing. According to previous studies, the relationship between the two types of information processing and task performance may be influenced by the degree of compatibility between the information processing characteristics and the task (Phillips et al. 2016), and the effect of analytic processing priming on creativity may vary depending on the task type. Analytic processing is controlled, sequential, and based on logic and rules, and may be more consistent with convergent than divergent thinking tasks. Studies have shown that performance on the CRT task, which reflects individuals’ analytic processing ability, correlates with performance on single-solution tests (e.g., fluid intelligence, statistical numerical reasoning, working memory, and mechanical-spatial ability) (Sobkow et al. 2022; Otero et al. 2022), and these results also provide supportive evidence for a possible relationship between analytic processing and convergent thinking. Thus, priming analytic processing could lead individuals to invest more cognitive effort and engage in more careful and conscious processing (Swami et al. 2014), resulting in better performance on convergent thinking tasks that require a single answer. However, the focused attention, linear processing, and slow processing speed associated with analytic processing could be detrimental to creative performance on divergent tasks (Baird et al. 2012). Therefore, Hypothesis 1 is proposed in the present study: there are differences in the effects of priming analytic processing on different types of creativity, particularly enhancing divergent thinking performance and decreasing convergent thinking performance.

In addition, thinking style could also be an important moderating variable in the relationship between analytic processing and creativity. Numerous studies have shown that individuals’ traits, tendencies, and styles can influence their psychological and behavioral performance, along with temporary priming manipulations, such as trait power and state power (Schmidt-Barad and Uziel 2020), trait mindfulness and state mindfulness (Egan et al. 2018), trait attachment and attachment priming (Cuyvers et al. 2022), and chronic regulatory focus and situational regulatory focus, etc. (Krishna et al. 2021). Thinking styles reflect the relatively stable tendency of individuals to rely on the two processing modes, and they may interact with analytic processing priming. There is limited research examining the effects of thinking styles and contextual priming on individuals. Only Dane et al. (2011) showed that individuals exhibit higher levels of average and above-average creativity when asked to choose a problem-solving approach that differs from their inherent thinking style. Dane suggested that this may be because invoking the kind of processing that is not normally used helps individuals engage in breakthrough cognitive associations that lead to increased levels of creativity. However, in the Dane et al. study, only one divergent thinking task (idea generation task) was used, and manipulated information processing was divided into intuitive and analytic processing priming groups without specifying a control group, making it difficult to assess the specific effects of analytic processing separately. Therefore, the moderating effect of thinking style on the relationship between analytic processing priming and the two types of creativity needs further investigation.

Individuals’ long-term preference for a particular type of cognitive processing may have increased the accessibility of this processing mode, such that it is often in a preporatory state in information-processing scenarios (Fang et al. 2018). From this perspective, information processing is more likely to be activated quickly and effectively when the priming effect matches the individual’s inherent thinking style, resulting in a higher priming effect. Therefore, thinking style and information processing priming may have an “additive effect” on creativity. Based on the above analysis, this study proposes Hypothesis 2: individuals’ thinking styles (experiential vs. rational) will moderate the predictive effect of analytic processing activation on divergent and convergent thinking performance; specifically, the predictive effect of analytic processing activation on both types of creativity will be stronger for individuals with high rational thinking style and individuals with low experiential thinking style.

The purpose of this study was to investigate the influence of analytic processing on performance on divergent and convergent thinking tasks and their boundary conditions in two experiments. Study 1 examined how analytic processing priming affects individual performance on two types of creative tasks (convergent and divergent) using a visual priming paradigm. In Study 2, we further examined how thinking styles moderate the influence of analytic processing priming on the two types of creativity.

## 3. Study 1

In this study, we examine how temporal priming of analytic processing influences divergent and convergent thinking performance. The visual priming paradigm was used to prime individual analytic processing to examine causal relationships between variables.

### 3.1. Participants and Procedure

The expected sample size was calculated using G*Power 3.1. Using *d* = 0.50 (a medium effect size), α = 0.05, and 1 − β = 0.80, it was calculated that at least 128 subjects were required. A total of 203 college students were recruited online to participate in the experiment, excluding those who did not complete the experiment and had previously participated in a similar experiment. The actual effective number of participants was 155, including 37 males and 118 females with a mean age of 22.11 years, *SD*_age_ = 2.21.

A single-factor within-subjects design (analytic processing group vs. control group) was adopted, in which subjects were randomly assigned to an analytic processing group (*n* = 77) or a control group (*n* = 78), with the analytic processing group priming analytic processing and the control group performing unrelated priming. After the priming manipulation, subjects completed two creative tasks, divergent and convergent, and completed the manipulation check items. The order of the divergent and convergent thinking tasks was counterbalanced between subjects to prevent the order of the processing of the creativity tasks from affecting the results.

### 3.2. Materials

#### 3.2.1. Analytic Processing Prime 

According to Gervais and Norenzayan (2012), a visual priming paradigm was used to activate participants’ analytic processing. The experimental group was asked to look at a picture of Rodin’s *The Thinker*, which shows a man sitting on a rock and thinking. The control group was asked to view a picture of Myron’s *Discobolus*, which shows a man throwing a discus. The viewing time was 30 s for both groups. Previous studies have shown that the image of *the Thinker* can effectively implicitly activate analytic processing (Franks and Scherr 2017).

#### 3.2.2. Divergent Thinking Task 

The Alternative Uses Test (Abbreviated as AUT, Guilford 1967) was used to assess individual performance in divergent thinking. Subjects were asked to list as many uses for the “rope” as possible within three minutes. Creativity scores were measured using three indicators: Fluency, Flexibility, and Originality. Fluency was assessed by the total number of ideas generated by a subject, and flexibility by the number of categories included in the ideas. Originality was assessed by the degree of uniqueness of an idea. The score was 0 if the frequency of an opinion was above 3%, 1 point if it was between 1% and 3%, and 2 points if it was below 1%. Considering that originality scores may be highly correlated with fluency scores (Nusbaum and Silvia 2011), we used a corrective calculation for originality (originality_corrected_ = originality/frequency) with reference to Shah and Gustafsson (2021).

#### 3.2.3. Convergent Thinking Task 

A validated Chinese version of the Remote Associates Test (RAT) (Xiao et al. 2016) was used to assess convergent thinking performance. Three words were assigned to each question. Subjects were asked to think of a word related to all three words (e.g., freshness, atmosphere, purification, and the answer word was air) within 3 min. The higher the score, the higher the level of convergent thinking. In this study, 14 of the 91 items from RAT were used, and this Chinese version was used among Chinese university students (Han et al. 2018; M. Zhang et al. 2020). 

#### 3.2.4. Manipulation Check 

To assess the effectiveness of the prime of analytic processing, each subject had to complete a short five-question scale to evaluate the procesing mode used in the creativity task (Dane et al. 2011). A 5-point Likert scale (1 = strongly disagree, 5 = strongly agree) was used with questions such as “I analyzed all available information in detail.” The higher the score, the more analytic processing was used in the task.

### 3.3. Results and Discussion 

#### 3.3.1. Manipulation Check 

The results of the independent samples *t*-test showed that participants reported higher use of analytic processing in the analytic processing group (*M* = 2.80, *SD* = 0.44) than in the control group (*M* = 2.59, *SD* = 0.49), *t*(153) = 2.81, *p* = 0.006, Cohen’s *d* = 0.45, suggesting a valid experimental manipulation of analytic processing.

#### 3.3.2. Hypothesis Testing

The descriptive statistical results of creativity task scores under different conditions are shown in Table 1. For the AUT performance, the results of the independent samples t-test showed that the analytic processing group scored significantly lower than the control group on fluency (*t*(153) = −2.51, *p* = 0.013, Cohen’s *d* = −0.41) and flexibility (*t*(153) = −2.46, *p* = 0.015, Cohen’s *d* = −0.40), but not on originality (*t*(153) = −0.74, *p* = 0.463, Cohen’s *d* = −0.12).

As for the RAT performance, results showed that the analytic processing group scored significantly higher than the control group on RAT scores (*t*(153) = 3.33, *p* = 0.001, Cohen’s *d* = 0.54).

To further exclude the possible effects of age and sex on the results, an ANCOVA was conducted with age and sex as covariates. The results showed a significant main effect of priming analytic processing on RAT scores (*F*(1, 151) = 10.94, *p* = 0.001, η_p_^2^ = 0.07), fluency (*F*(1, 151) = 6.09, *p* = 0.015, η_p_^2^ = 0.04), flexibility (*F*(1, 151) = 6.12, *p* = 0.014, η_p_^2^ = 0.04), but not on originality (*F*(1, 151) = 0.55, *p* = 0.459, η_p_^2^ = 0.004). 

Meanwhile, no significant effect was found for gender and age on the four variables (AUT-fluency, AUT-flexibility, AUT-originality, and RAT), *F*s < *1.00*, *p*s > 0.05. 

The results were consistent with Hypothesis 1 that there were differences in the effects of analytic processing on performance on different types of creative tasks. Specifically, priming analytic processing significantly increased the RAT performance and decreased the fluency and flexibility scores of AUT compared to the control group.

## 4. Study 2

Based on the results of Study 1, Study 2 introduced the measurement of experiential and rational thinking styles to examine how the analytic processing prime and individual inherent thinking styles affect individual performance on the two types of creative tasks. In addition, to improve the robustness of the results of Study 1, the implicit verbal fluency task-priming paradigm was used to activate analytic processing.

### 4.1. Participants and Procedure

The sample size required for the study was calculated using G*Power3.1, and given the need for a moderating effect analysis, *F* tests (linear multiple regression: fixed model, *R*^2^ increase) were chosen, setting the effect size *f*^2^ = 0.10, α = 0.05, and 1 − β = 0.80, to calculate the minimum 100 subjects required. A total of 134 college students were recruited online to participate in the experiment, excluding those who did not complete the experiment seriously and those who had completed similar questions. The final valid sample size was 119. Of those, 58 subjects were included in the control group and 61 in the analytic processing group. There were 20 males and 99 females, with a mean age of 21.82 (*SD* = 2.06).

Identical to Study 1, a one-factor between-subjects experimental design was used, with analytic processing priming in the experimental group and irrelevant priming in the control group. First, subjects were required to fill out a questionnaire measuring experiential-rational thinking style, after which they were randomly assigned to either the experimental or control group, and after priming was completed, subjects completed two creativity tasks of divergent and convergent as well as manipulation check items.

### 4.2. Materials

#### 4.2.1. Analytic Processing Prime 

Following Gervais and Norenzayan’s (2012) implicit priming paradigm, word selection and sentence formation tasks were used to activate analytic processing. The task consisted of ten groups of five words each. Subjects had to exclude one word and rearrange the remaining four words to form a complete sentence (e.g., five words: love, my, run, I, parents; target sentence: I love my parents). In each word group, the excluded word was the target word used to manipulate processing. In the analytic processing group, five excluded words were related to analytic reasoning (e.g., “think,” “rational”), whereas in the control group, all 10 excluded words were conventional words unrelated to analytic reasoning. The effectiveness of this priming paradigm for activating analytic processing has been verified in a previous study (Geng et al. 2020), that is, analytic processing can be activated concomitantly through the initiation of trait construct.

#### 4.2.2. Thinking Style Measurement 

The Rational-Experiential Inventory for Adolescents (REI-A, Marks et al. 2008) was used to measure individual thinking style, which includes two dimensions of experiential and rational thinking styles, with 10 questions for each dimension (e.g., I try to avoid situations that require thinking in depth about something), through a 5-point Likert scale, ranging from 1 (completely inconsistent) to 5 (completely consistent). Cronbach’s α for the experiential and rational thinking style subscales in this study were 0.79 and 0.86, respectively.

#### 4.2.3. Divergent Thinking Task, Convergent Thinking Task, and Manipulation Check 

The divergent thinking task, convergent thinking task, and manipulation check items applied in study 2 were identical to Study 1.

### 4.3. Results and Discussion 

#### 4.3.1. Manipulation Check 

First, an independent sample *t*-test was conducted to examine whether there was a significant difference between the analytic processing group and the control group in the manipulation test. The results showed that the analytic processing group (*M* = 2.85, *SD* = 0.51) reported a higher use of analytic processing than the control group (*M* = 2.65, *SD* = 0.42), *t*(121) = 2.29, *p* = 0.024, Cohen’s *d* = 0.42; thus, the manipulation was effective.

#### 4.3.2. Hypothesis Test 

To investigate the combined influence of individual inherent thinking styles and analytic processing priming on convergent and divergent thinking, a multiple regression equation was constructed (see Table 2). First, the priming condition was coded as a virtual variable (0-control group, 1-analytic processing group), and all continuous variables included in the regression equation were standardized. Simultaneously, the standardized score for the rational and experiential thinking styles is multiplied by the primed condition to form an interactive term. The score of the convergent thinking task (RAT) and three indicators of the divergent thinking task (fluency, flexibility, and originality) were taken as dependent variables, the priming conditions and rational and experiential thinking styles were added as predictive variables in Model 1, and two interaction items were added to Model 2. 

For convergent thinking performance, Model 1 results showed that the priming condition positively predicted RAT scores after controlling for experiential thinking style and rational thinking style scores (*B* = 0.43, *t* = 2.35, *p* = 0.021); that is, priming analytic processing facilitated individual RAT performance compared to the control group. In Model 2, neither interaction terms were a significant predictor of RAT performance (*p* > 0.05).

For the fluency score of AUT, Model 1 results showed that the predictive effect of the priming condition on fluency score was significant (*B* = −0.37, *t* = −2.00, *p* = 0.048), and the predictive effects of both experiential and rational thinking styles were not significant (*p*s > 0.05). In Model 2, the interaction term between analytic processing priming and rational thinking style negatively predicted fluency score (*B* = −0.42, *t* = −2.36, *p* = 0.020), suggesting that rational thinking style significantly and negatively moderated the predictive effect of the priming condition on AUT-fluency score.

For the flexibility score, Model 1 results showed that the predictive effects of the priming condition on the flexibility score reached marginal significance (*B* = −0.34, *t* = −1.83, *p* = 0.070), and the predictive effects of both experiential and rational thinking styles were not significant (*p*s > 0.05). In Model 2, the interaction term between analytic processing priming and rational thinking style negatively predicted flexibility score (*B* = −0.53, *t* = −2.93, *p* = 0.004), suggesting that rational thinking style significantly negatively moderated the predictive effect of the priming condition on AUT-flexibility score.

For the originality score, Model 1 results showed that the predictive effects of the priming condition on the originality score was not significant (*B* = −0.28, *t* = −1.50, *p* = 0.136). In Model 2, the interaction terms of analytic processing priming with experiential and rational thinking styles were not significant predictors of AUT-originality score (*p*s > 0.05).

**Figure 1:**
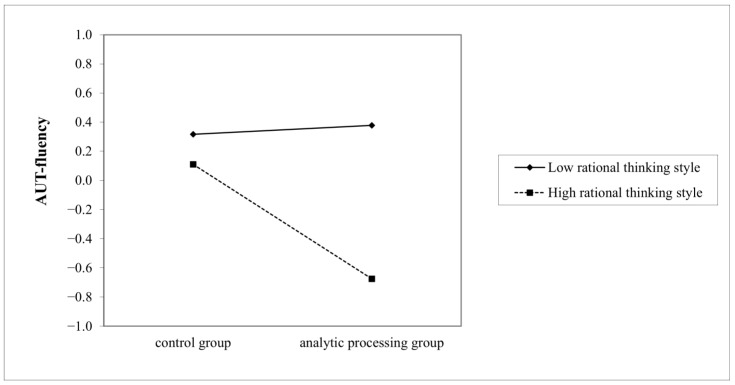
The interaction between rational thinking style and analytic processing prime on fluency.

**Figure 2:**
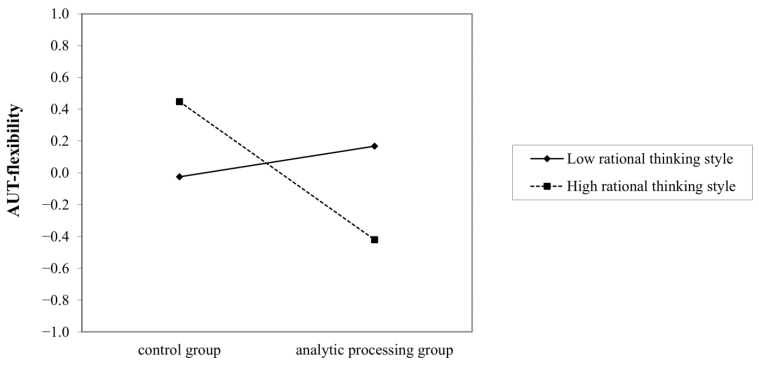
The interaction between rational thinking style and analytic processing prime on flexibility.

Further, a simple slope analysis was conducted to clarify the moderating role of the rational thinking style. Among individuals with a high rational thinking style (*M* + *SD*), analytic processing priming had a significant negative predictive effect on AUT-fluency (*B* = −0.79, *t* = −3.11, *p* = 0.002), whereas, among individuals with a low rational thinking style (*M* − *SD*), the predictive effect of priming was not significant (*B* = 0.06, *t* = 0.24, *p* = 0.810) (see Figure 1). Similar results were found for flexibility score, that among individuals with a high rational thinking style (*M* + *SD*), analytic processing priming had a significant negative predictive effect on AUT-flexibility (*B* = −0.87, *t* = −3.38, *p* = 0.001), whereas among individuals with a low rational thinking style (*M* − *SD*), the predictive effect of priming was not significant (*B* = 0.19, *t* = 0.75, *p* = 0.456) (see Figure 2).

## 5. General Discussion 

Through two experiments, this study examined how priming analytic processing affects people’s performance on both divergent and convergent thinking tasks and how individuals’ inherent thinking styles moderate this effect. 

This study found significant differences in the effects of priming analytic processing on different types of creativity. For convergent thinking, subjects in the analytic processing-primed group performed better on the RAT task than the control group, which is consistent with the findings of Barr et al. (2015). Convergent thinking task requires the generation of relevant answers based on well-defined criteria, relies on top-down cognitive processing (Jiang et al. 2022; Hommel et al. 2011), and requires individuals to invest a high level of cognitive resources to exercise cognitive control and prevent irrelevant ideas from entering the working memory (Radel et al. 2015). This process is closely linked to conscious, mental resource-draining, and logically reasoned analytic processing. Thus, priming analytic processing may help individuals stay focused during convergent thinking tasks, perform careful search and logical analysis, and eliminate distractions to find a single, correct solution.

For divergent thinking, the results showed that the analytic processing priming group performed lower fluency and flexibility scores than the control group, in line with Deshayes et al. (2021). The divergent thinking task process allows people to generate as many responses (ideas, solutions) as possible under relatively weak constraints, and under this premise, lower levels of cognitive control may facilitate individuals to quickly “jump” from one idea to another (Hommel et al. 2011) and help allow more unfiltered information to enter the processing stage, thereby facilitating the generation of more divergent ideas (Radel et al. 2015). Consequently, automatic, associative, and rapid intuitive processing may yield more benefits when performing divergent thinking tasks than slow, highly controlled analytic processing (Norris and Epstein 2011). Based on the dual processing theory perspective, when analytic processing is activated and cognitive resources are abundant, intuitive processing is often overridden (Strack and Deutsch 2004); therefore, activating analytic processing may limit individuals’ intuitive processing in divergent thinking tasks and reduce the number and categories of the generated ideas. In addition, Beaty et al. (2019) suggested that during creative cognition, Default Network (DN, supporting spontaneous thought) might get involved in idea generation and the Executive control network (ECN, supporting cognitive control) in guiding, constraining, and modifying DN processes. Razumnikova and Yashanina (2017) found that rational thinking and irrational thinking were associated with the frequency-regional organization of cortical alpha range biopotentials in convergent and divergent tasks, respectively. These results also provide cognitive-neural evidence to support the differential effects of analytic processing on divergent and convergent thinking task performance found in this study. However, the numerical difference in the originality score between the analytic processing group and the control group did not reach significance, which might be due to the task duration. Within 3 min, some particularly novel ideas may have not been fully explored, resulting in the non-significant difference in originality between the two groups. Alternatively, analytic processing might make participants evaluate the generated ideas based on the task constraints (Palmiero et al. 2020), and eliminate the more “common” answers during the idea generation process, thus preserving originality at the expense of fluency and flexibility. 

In the present study, it was found that there was a boundary condition for the impairment of fluency and flexibility of AUT task by analytic processing priming; that is, the effect was only present in individuals with a high propensity for rational thinking, indicating that contextual analytic processing priming and rational thinking style, rather than experiential thinking style, synergistically influence divergent thinking performance, thus, Hypothesis 2 was partially tested. The findings also partially support Dane et al.’s (2011) finding that priming of analytic problem solving is detrimental to the creative performance of individuals with high rational thinking styles. This may be because priming is a concomitant activation of knowledge structures such as characteristic concepts or habits (Bargh et al. 1996), and individuals with high rational thinking have a long-term, stable preference for using analytic processing compared to individuals with low rational thinking style (Norris and Epstein 2011), therefore, their analytic processing systems may be more accessible and more likely to be activated quickly and effectively at the time of priming, and thus, be more susceptible to the effects of analytic processing priming, showing a decrease in divergent creativity. For convergent thinking task scores, thinking styles did not interact with analytic processing priming, possibly because the convergent thinking task itself has higher demands on analytic processing. According to previous research, the effect of information processing priming on task performance may be stronger when task features are higher in matching compatibility with information processing type (Phillips et al. 2016; Rusou et al. 2013); thus, even individuals with low rational thinking may be susceptible to analytic processing priming and show enhanced task performance in convergent thinking tasks. It is worth noting that no moderating effect of experiential thinking style was found in the present study, which is also consistent with Dane et al. (2011), possibly because the experiential thinking style and rational thinking style are independent of each other (Marks et al. 2008). Therefore, the level of an individual’s experiential thinking style does not reflect his or her preference for the use of analytic processing, nor does it interact directly with analytic processing priming.

This study advances theoretical and empirical research in the following ways. First, although dual process theory has accumulated a substantial theoretical and empirical foundation in both situational and dispositional research orientations (Gervais and Norenzayan 2012; Epstein et al. 1996), few studies have combined the priming of analytic processing with thinking styles to examine its effects on individuals. Within the framework of dual process theory, the present study systematically discusses the role of a stable thinking style and states activation of analytic processing on two creativity task performances, to some extent, adds to the study of creativity from the perspective of dual process theory. Second, by adopting AUT and RAT as creativity measures, this study indicates that analytic processing may have different effects on the two important components of creativity, divergent and convergent thinking, providing a probable perspective to explain the differences in the results of previous studies. Additionally, the research findings offer implications for the cultivation of creativity in education, organization, and other fields. Relevant practitioners should pay attention to the differentiated impact of analytic processing on different types of creativity. The results suggest that the priming or intervention of analytic processing could serve as a possible strategy for enhancing creativity, but it may play a greater role in creative tasks or phases that are dominated by convergent rather than divergent thinking. In addition, the potential influence of individual differences should be taken into account in analytic-processing-related-creativity promotion programs, and more effective interventions should be developed in conjunction with individual thinking styles.

This study has several limitations. First, we adopted two widely used creative tasks in the present study, however, there are numerous measures of divergent and convergent thinking, such as insight problems, Evaluation of Creative Potential (EPoC), etc. (Barbot et al. 2016). Therefore, it is necessary to examine the robustness of the results by applying multiple measures of creativity in future research. At the same time, there is a discrepancy between the cognitive creativity task under experimental conditions and the real innovation process, so in the follow-up studies, empirical sampling and other approaches could be considered to investigate the role of intuitive and analytic processing in the real innovation process. Second, the subjects in this study were all college students, which may limit the generalizability of the results. In the future, relationships between the variables in different groups will be discussed. Third, this study did not investigate the mechanism of analytic processing that influences creativity. In future research, neuroscience or other experimental methods could be used to further investigate the underlying mechanism of its effect on creative cognitive process.

## 6. Conclusions

Overall, through two experiments, the present study examined the effects of analytic processing priming on divergent and convergent thinking task performance and the moderating effects of individual thinking styles from the perspective of dual process theory. The results indicated that priming analytic processing enhanced individuals’ performance on the convergent thinking task (RAT) and reduced individuals’ fluency and flexibility scores on the divergent thinking task (AUT). Furthermore, the negative effects of analytic processing priming on AUT fluency and flexibility were significant only in individuals with a high rational thinking style.

## Figures and Tables

**Table 1:** Creativity task scores under different conditions (*M* ± *SD*).

	AUT			RAT
	Fluency	Flexibility	Originality	
control group (*n* = 78)	8.10 ± 1.88	5.32 ± 1.54	0.18 ± 0.22	6.47 ± 2.41
analytic processing group (*n* = 77)	7.29 ± 2.17	4.74 ± 1.39	0.15 ± 0.17	7.82 ± 2.61

**Table 2:** Moderating effects of experiential and rational thinking styles.

Predictor	RAT		AUT-Fluency		AUT-Flexibility		AUT-Originality	
	Model 1	Model 2	Model 1	Model 2	Model 1	Model 2	Model 1	Model 2
Condition	0.43 *	0.43 *	−0.37 *	−0.36 *	−0.34 ^+^	−0.34 ^+^	−0.28	−0.28
Experiential thinking style	−0.12	−0.05	0.12	0.15	0.01	0.03	−0.10	−0.05
Rational thinking style	0.03	−0.01	−0.10	0.10	−0.02	0.24^+^	0.10	0.25^+^
Condition * experiential thinking style		−0.15		−0.07		−0.03		−0.02
Condition * rational thinking style		0.10		−0.42 *		−0.53 **		−0.31 ^+^
*R* ^2^	0.07	0.08	0.07	0.11	0.03	0.10	0.03	0.06
*F*	2.83 *	1.87	2.72 *	2.85 *	1.22	2.52 *	1.25	1.41
Δ*R*^2^		0.01		0.05 ^+^		0.07 *		0.03

Note: * *p* < 0.05, ** *p* < 0.01, ^+^ *p* < 0.1, Non-standardized coefficient *B* was reported in this table. The primed condition was a dummy-coded predictor, where 0 = control group and 1 = analytic processing group.

## Data Availability

The data presented in this study are available on request from the corresponding author.
